# Supplementary material for: Complexome profiling of the Chlamydomonas psb28 mutant reveals TEF5 as an early PSII assembly factor
Source: Plant Cell. 2025 Mar 17;37(6):koaf055. doi: 10.1093/plcell/koaf055 (PMC12164590; doi:10.1093/plcell/koaf055)
Supplement: koaf055_Supplementary_Data [file koaf055_supplementary_data.zip › psb28_interactive.zip!/plots/intensity/qualityPlots/peptides/MME6_Cre06.g251400.t1.2.html]

### Cre06.g251400.t1.2

Trivial name: MME6  
SeqCoverage [median]: 23.70  
Mass: 67.59 kDa  
  
 

| Sequence | MissedCleavage | Mass [Da] | wtIntensity | muIntensity | Score |
| --- | --- | --- | --- | --- | --- |
| ALVATEQR | 0 | 886.49 | 3158.16 | 2554.66 | 128.86 |
| AYEGGFATALPKPHNLYEAAR | 1 | 2275.14 | 0.00 | 5735.63 | 53.28 |
| GLAFSDAERDR | 1 | 1235.59 | 0.00 | 1278.48 | 34.72 |
| GLFITLEDR | 0 | 1062.57 | 1013.35 | 0.00 | 95.53 |
| LLVVSDGER | 0 | 986.54 | 1982.67 | 7155.10 | 96.25 |
| LRDEQFIAAADAVSR | 1 | 1660.85 | 1271.58 | 3762.95 | 78.93 |
| LVGEEDLAR | 0 | 1000.52 | 3765.05 | 2877.95 | 72.48 |
| QSMYHPQYR | 0 | 1208.54 | 406.67 | 417.28 | 60.02 |
| QVISGVDLMR | 0 | 1116.60 | 0.00 | 656.24 | 85.68 |
| STYMQSLQER | 0 | 1241.57 | 0.00 | 445.76 | 76.83 |
| VGDLGDLGVQAVGVPISK | 0 | 1722.95 | 2920.57 | 0.00 | 153.81 |
|  |  |  | 14518.0 | 24884.1 |  |
